# Pediatric Asthma Action Plans: National Cross-Sectional Online Survey of Parents' Perceptions

**DOI:** 10.2196/21863

**Published:** 2020-11-09

**Authors:** Karen H Pletta, Bradley R Kerr, Jens C Eickhoff, Gail S Allen, Sanjeev R Jain, Megan A Moreno

**Affiliations:** 1 Department of Pediatrics University of Wisconsin-Madison Madison, WI United States; 2 Department of Biostatistics & Medical Informatics University of Wisconsin-Madison Madison, WI United States

**Keywords:** pediatric asthma, asthma action plan, parent, online survey, self-efficacy, daily living factors, parental work, school absence, school management, caregiver management, child activity, primary care provider, pediatrician, asthma specialist

## Abstract

**Background:**

Asthma Action Plans (AAPs) are recommended for pediatric patients to help improve asthma control. Studies have shown variable results for unscheduled doctor and emergency room visits. AAPs may have an impact on parental self-efficacy for asthma management as well as on other daily living factors that are valuable for patients and families, such as the number of missed school days and parental workdays, and on school and caregiver management.

**Objective:**

The purpose of this study is to understand parent perceptions of AAPs. The goals of this analysis were threefold, including examining (1) the association between pediatric AAPs and parental self-efficacy, (2) parent perceptions of the helpfulness of an AAP for daily living factors, and (3) associations with the type of provider who gave the AAP (a primary care provider or an asthma specialist).

**Methods:**

A national cross-sectional online survey was completed in October 2018 by parents of children with asthma aged 0-17 years. Survey questions included the presence or absence of a pediatric AAP, the Bursch Parental Self-efficacy for Asthma scale, parental perceptions of the AAP's helpfulness with regard to daily living factors ranked on a 5-point Likert scale, and the provider type who gave the AAP. Survey responses were summarized in terms of percentages or means and standard deviations. A 2-sample *t* test and analysis of covariance were used to compare self-efficacy for asthma and parental-perception-of-helpfulness scores between subjects with an AAP versus subjects without an AAP. All reported *P* values were 2-sided.

**Results:**

A total of 704 parents with a child with asthma completed the survey. The parents had a mean age of 37.5 years (SD 10.9), and 82% (577/704) were women and 18% (127/704) were men. Most (564/704, 80%) parents had an AAP for their child; 65% (367/564) were written, 51% (286/564) were online, and 84% (474/564) were available at school. The Bursch Self-efficacy scale was significantly higher for parents with an AAP (mean 57.7, SD 8.6) versus no AAP (mean 55.1, SD 9.9; *P*<.001). Parents reported that they agreed/strongly agreed that an AAP was helpful for daily living factors, including managing asthma (446/544, 82%), decreased parental missed workdays (367/544, 68%), decreased child missed-school days (396/542, 73%), and for when a child is at school (422/541 78%), with other caregivers (434/543, 80%), doing normal activities (421/540 78%), and leading a normal life (437/540 81%). Parents agreed/strongly agreed that an AAP was helpful from all provider types: a pediatric provider (583/704, 82.8%), a family practice provider (556/704, 79%), and an asthma specialist (594/704, 84.4%). There was no significant difference (*P*=.53) between the type of provider who gave the AAP.

**Conclusions:**

Parents who had pediatric AAPs for their children reported increased parental self-efficacy compared to those who did not have AAPs. Parents found AAPs helpful for decreasing missed time from work and school, and for asthma management when at home, school, and with other caregivers. Significant AAP helpfulness was seen regardless of the provider who gave the AAP, the parent's education, and income level. Findings support the usefulness of pediatric AAPs for families and the development of easily sharable electronic AAPs for children.

## Introduction

### Background

Asthma is the most common chronic condition of childhood [1], affecting 7 million children in the United States [2]. Pediatric asthma can result in severe breathing distress [3], causes over a half million hospitalizations each year, and is the leading reason for hospitalization for children aged 1-17 years [4]. Further, caring for a child with asthma can lead to negative impacts on daily living for patients and families. Factors of daily living that may be negatively impacted by asthma include children missing days from school, parents missing days from work, and challenges in coordinating care with caregivers and schools [5].

To reduce health risks and quality of life impairments associated with asthma, the Centers for Disease Control and Prevention and the American Academy of Pediatrics recommend providing pediatric asthma patients with an Asthma Action Plan (AAP). An AAP is a written plan for the daily control of asthma symptoms, including steps for a family to follow if asthma symptoms develop or worsen [6,7]. The AAP may be on paper, online, or both, and can be provided to a family by a primary care provider (PCP) or an asthma specialist as part of routine asthma care. An AAP is intended to increase child and parental knowledge as well as child self-efficacy and parental self-efficacy (ie, parental perception of one’s ability to provide appropriate care) in managing the child's asthma. Parents and children are instructed about AAPs during clinic visits with older children and adolescents who are increasingly managing their symptoms independently. Thus, an AAP may lead to better control of asthma symptoms, reduce unplanned medical visits, and improve the consistency of asthma care that children receive at school and with caregivers.

Despite these potential benefits, studies measuring the impact of AAPs on asthma complications have shown variable results. Some studies have not supported that providing AAPs to children is associated with a decrease of symptom-free days, urgent care visits, or unscheduled doctor visits [8-10], while other studies on AAP use found a decrease in missed school days, emergency room use, and unscheduled doctor visits [11-13], as well as improved confidence in parents caring for children with asthma [11]. AAP effectiveness may be influenced by income and literacy. There are several factors that may create challenges for those with lower income and asthma, including access to medical care, an urban environment, and lower literacy [14]. Lower-income families are less likely to have an AAP [14] and have an increased risk for asthma treatment failure and exacerbations [15]. Low literacy has been linked to misunderstandings of AAP medications and instructions [16], as well as greater asthma severity [17]. Thus, additional research is needed to elucidate the role of AAPs in reducing asthma complications.

Pediatric asthma exacerbations can negatively impact daily living factors for pediatric patients and families. These factors may be influenced by parental asthma self-efficacy, parental perception of AAP helpfulness, and which type of provider supplies the AAP. Parental asthma self-efficacy may influence whether parents feel able to take steps at home to manage their child's asthma. Further, the perception of AAP helpfulness may influence whether parents decide to use the AAP in the event of an asthma exacerbation, and the perception may vary depending on income and college education. Finally, patients may be provided an AAP by their PCP or an asthma specialist. Referral to an asthma specialist may depend on several factors, including the PCP’s comfort level with managing asthma, the severity of asthma, and limitations of access and geography. Parents may view the AAP differently if provided in a primary-care versus specialty setting.

### Objectives

The purpose of this study is to understand parent perceptions of pediatric AAPs. The goals of this analysis were threefold, including examining (1) the association between pediatric AAP provision and parental asthma self-efficacy, (2) parents' perceptions of the helpfulness of an AAP for daily living factors, and (3) associations with the type of provider who gave the AAP (PCP or asthma specialist).

## Methods

In October 2018, we conducted a cross-sectional survey using a national online panel (Qualtrics; version 102018); the Institutional Board at the University of Wisconsin-Madison approved this study.

### Participants and Recruitment

We recruited parents using Qualtrics. Qualtrics was chosen as the platform because surveys deployed to Qualtrics panels allow for a focus on participants meeting specific criteria. The online survey platform allowed for the identification of parents and, particularly, the oversampling of children with chronic diseases. Further, Qualtrics panels typically demonstrate demographic characteristics that fall within a 10% range of values observed in the US population [18]. Qualtrics recruits participants using online advertisements on platforms such as social media, inviting survey participants to earn credit toward rewards such as gift cards, in-application purchases, or airline miles. A background check is conducted to verify identity before the participant becomes part of a panel and eligible for recruitment.

Qualtrics sent survey invitations to existing US panel members who were English-speaking parents of children 0-17 years old in order to obtain a group consisting of 25% of parents with a child with a chronic illness. This asthma analysis was part of a larger survey of 3000 parents. The design was planned to include at least 25% of parents with children with chronic disease toward appropriate sample sizes for studies of children with and without chronic disease. A recruitment message was emailed to potentially eligible individuals notifying them of a survey opportunity, describing the estimated survey length (15 minutes), and informing them that up to $20 in e-Rewards credit could be obtained in return for participation. All participants provided informed consent. Parents who noted that their child had asthma were included in analyses.

### Survey Measures

Demographic variables included age, gender identity, race, ethnicity, parental education level, household income, residential setting (rural, suburban, or urban), and geographic region (Midwest, Northeast, South, West) [19].

### Asthma Action Plan Presence

Participants were asked if they had an AAP for their child. If they did, they were asked if the AAP was written, online, and available at their school (“yes” or “no”). Participants were then asked which type of provider gave the AAP to the parent. Response options included “pediatric provider,” “family practice provider,” “asthma specialist,” or “other.” Responses were mutually exclusive.

### Parental Asthma Self-Efficacy

Parental asthma self-efficacy was assessed using the Bursch Parental Self-efficacy for Asthma scale [20]. This survey includes 13 questions such as “How sure are you that you would know which medications to use when your child is having a serious breathing problem?” and “How sure are you that you can help your child to prevent a serious breathing problem?” Participants provided responses using a 6-point Likert scale, from 1 = “Not at all sure” to 5 = “Completely sure” and 6 = “Does not apply,” per the Bursch Parental Self-efficacy scale protocol. The total score ranged from 13 to 65, with a higher score indicating a higher level of self-efficacy.

### Parent Perceptions of Helpfulness of the AAP for Daily Living Factors

Participants were asked to indicate their perceptions of the AAP with regard to its helpfulness for daily living factors by indicating their agreement with a series of statements. These included a general statement, “the Asthma Action Plan has been helpful for managing my child's asthma,” as well as statements for specific factors, such as “the Asthma Action Plan decreases the number of days that I miss work due to my child's asthma,” “the Asthma Action Plan decreases the days that my child misses school due to asthma,” “the Asthma Action Plan is helpful for when my child is at school,” and “the Asthma Action Plan is helpful when my child is with another caregiver.” Responses used a 5-point Likert scale, which ranged from 1 = “Strongly disagree” to 5 = “Strongly agree.”

### Analysis

Descriptive analyses were summarized in terms of means and standard deviations or proportions. For the helpfulness of daily living factors, results were combined to report on the proportion who agreed or strongly agreed and summarized in terms of frequencies and percentages. A 2-sample *t* test was used to compare self-efficacy for asthma and parental-perceptions-of-helpfulness scores between subjects with an AAP versus subjects without an AAP. An analysis of covariance (ANCOVA) was conducted to adjust for comparison by parents’ age, gender, education, household income, and provider type. All reported *P* values were 2-sided, and *P*<.05 was used to define statistical significance. Statistical analyses were conducted using SAS software (version 9.4; SAS Institute).

## Results

### Participants

A total of 704 parents reported having a child with asthma and completed the survey. Participants had a mean age of 37.5 (SD 10.9) years; 82.1% (578/704) were women and 17.9% (126/704) were men; 65% (458/704) had a college degree. The majority of participants (429/704, 61%) had an income below the national median (<$75,000), and 68.2% (480/704) lived in suburban or urban settings. Table 1 displays demographic data.

**Table 1:** Demographic data of the parent participants (n=704).

Participant characteristics		Values, n (%)
**Gender**		
	Male	122 (17.4)
	Female	575 (82.1)
**Race**		
	White	546 (77.5)
	Hispanic	111 (15.9)
	Black	80 (11.4)
	Asian	21 (2.9)
	American Indian	15 (2.1)
	Native Hawaiian	7 (1.0)
	Multiracial	24 (3.4)
**Education**		
	No college degree	238 (34)
	College degree	464 (65)
**Household income**		
	<$75,000	431 (61)
	>$75,000	272 (39)
**Residential setting**		
	Rural	223 (31.9)
	Suburban	291 (41.6)
	Urban	186 (26.6)
**Geographic region**		
	Midwest	163 (23.8)
	Northeast	135 (19.7)
	South	264 (38.5)
	West	124 (18.1)

### Asthma Action Plan Presence

Most participants (564/704, 80%) reported that they had an AAP for their child. AAPs were available to 65% (367/564) of participants in written form and 51% (288/564) online. Most participants (474/564, 84%) reported that the AAP was available at school.

### Association of AAP with Parental Asthma Self-Efficacy

Parental asthma self-efficacy was significantly higher for parents with an AAP (mean 57.7, SD 8.6) compared to parents who did not have an AAP (mean 55.1, SD 9.9, *P*<.001). After adjusting for parents’ age, education, household income, gender, and provider type, the adjusted mean for parents with an AAP was 58.3 (95% CI 52.2-64.4) versus 54.8 (95% CI 48.7-60.9) for parents without an AAP (p=0.0005).

### Helpfulness of the AAP for Daily Living Factors

Among the 564 parents who had an AAP, 82% (462/564) agreed or strongly agreed with the general statement, “the Asthma Action Plan has been helpful in managing my child’s asthma.” Most parents agreed or strongly agreed that having an AAP was helpful for individual daily living factors, including the management of asthma (446/544, 82%), decreased parental missed workdays (367/544, 68%), decreased child missed school days (396/542, 73%), and for times when the child is at school (422/541, 78%), with other caregivers (434/543, 80%), doing normal activities (421/540, 78%), and leading a normal life (437/540, 81%). Table 2 shows the participant ratings of the helpfulness of the AAP.

**Table 2:** Participant ratings of the helpfulness of Asthma Action Plans (AAPs).

Asthma management factors	N	Mean agreement rating^a^ of AAP helpfulness, Mean (SD)
Management of the child’s asthma	544	4.2 (0.9)
Comfort with managing the child’s asthma	541	4.2 (0.9)
Decrease in parental missed workdays	544	3.9 (1.1)
School asthma management	541	4.1 (1.0)
Decrease in the child’s missed school days	542	4.0 (1.0)
Caregiver asthma management	543	4.2 (0.9)
Ability for the child to have normal activity	540	4.1 (1.0)
Ability for the child to lead a normal life	540	4.1 (0.3)

^a^A Likert scale was used, ranging from 1=“Strong disagree” to 5=“Strong agree.”

### Helpfulness of the AAP and Provider Type, Household Income, and Education

The proportion of participants who reported agreement/strong agreement for the question “The Asthma Action Plan has been helpful in managing my child’s asthma” were compared between provider type (pediatric provider, family practice provider, and asthma specialist), household income (≥$75,000 versus <$75,000), and education level (college education versus no college education). There was no significant difference (*P*=.53) observed in the rates of participants who reported agreement/strong agreement for the question “The Asthma Action Plan has been helpful in managing my child’s asthma” when comparing between provider types: 83% (583/704) for a pediatric provider, 79% (556/704) for a family practice provider, and 84% (594/705) for an asthma specialist. For participants with a household income of ≥$75,000, the rate of participants who agreed/strongly agreed with the question “The Asthma Action Plan has been helpful in managing my child’s asthma” was 85% (598/704), as compared to 80% (563/704) for participants with a household income of <$75,000 (*P*=.20). Furthermore, there was no significant difference (*P*=.48) detected in the rates of participants who reported agreement/strong agreement with the question “The Asthma Action Plan has been helpful in managing my child’s asthma” between participants with a college degree or above (570/704, 81%) versus participants without a college degree (591/704, 84%). Table 3 displays the logistic regression analysis results for the prediction of whether the AAP is helpful at managing asthma.

**Table 3:** Results of the logistic regression analysis for predicting whether an Asthma Action Plan (AAP) is helpful for managing asthma.

Participant variable		Rate of respondents who answered “agree” or “strongly agree” to the question “AAP is helpful at managing my child’s asthma,“ % (95% CI)	Odds ratio (95% CI)	*P* value	
**Provider who gave AAP**					.53
	Pediatrics	82.8 (78.2%-87.3%)	0.89 (0.46-1.69)	.72	
	Family practice	79.0 (72.4%-85.6%)	0.69 (0.34-1.41)	.30	
	Asthma specialist	84.4 (76.5%-92.2%)	Reference		
**Household income**					
	≥ $75,000	84.5 (79.1%-90.0%)	1.41 (0.83-2.40)	.20	
	< $75,000	79.6 (74.2%-85.0%)	Reference		
**Parent education**					
	College degree	80.7 (74.5%-86.8%)	0.82 (0.48-1.42)	.48	
	No college degree	83.5 (78.6%-88.3%)	Reference		

## Discussion

### Principal Findings

This study sought to understand associations of pediatric AAP provision with parental self-efficacy for asthma, perceptions of the AAP’s helpfulness for managing daily living factors for families, and whether the helpfulness of the AAP differed based on the type of provider who gave the plan. We found that parents who had an AAP had higher parental self-efficacy than parents who did not have an AAP, and that most parents viewed the AAP as helpful for many daily living factors. Further, there was no difference in parental perceptions of AAP helpfulness, whether provided by a PCP or asthma specialist.

Our first finding was that parents who had AAPs had slightly, but statistically significant, higher asthma parental self-efficacy than parents who did not have AAPs. Our finding is consistent with a recent study suggesting that AAP use is associated with improved confidence for parents caring for children with asthma [11]. The asthma self-efficacy questions included measures for comfortableness for, understanding of, and feelings of the ability to care for a child’s asthma symptoms, which can contribute to confidence. Taken together, these studies support that AAPs can lead to (in the very least, small) improvements in parents’ abilities to care for their children with asthma. Of note, all our participants, both with and without an AAP, had high mean scores, so there may have been a ceiling effect. As the surveyed parent population participated in the study via the Qualtrics format, they had access to, and the ability to use, the internet, and they may have used the internet to access supportive health information. Thus, they may have higher self-efficacy than parents who are not able to use or do not have internet access. Further investigation of AAP experiences among parents with limits on internet use or access is warranted.

Our second finding was that most parents perceived the AAP to help daily living factors, regardless of income and education. Parents perceived that AAP use helped decrease their time missed from work and their child’s time missed from school due to asthma. This contrasts with a finding in which parents who used AAPs did not report a significant decrease of child symptom-free days compared to parents who did not use an AAP; those authors considered that perhaps AAP users have more symptoms and, thus, worse outcomes [8]. However, another study found that AAP use was associated with reduced school absence days due to asthma [11]. Our findings of parents’ perceptions of a decreased need for work absences and school absences is consistent with this; it may indicate that AAP use decreased the severity of symptoms in at least some children, such that time away from work and school were reduced. Overall, this analysis of parent perceptions of AAP helpfulness for daily living factors supports that parents perceive benefits from having a plan and a sense of control for managing their child’s asthma. Areas that involve an easing of child care, such as management during school time and time with caregivers, may be important to maintaining regular daily activities. AAPs may be useful for others beyond parents, helping teachers, school personnel, and other caregivers during school, daycare, and after-school activity hours. Further studies directly assessing asthma control and AAP use by parents, school personnel, and caregivers would be valuable.

Finally, a high percentage of parents reported the AAP to be helpful regardless of the provider who distributed the plan. These findings were consistent even with adjustments for income and education. This result implies that an AAP can be a beneficial tool for children with asthma when distributed through an asthma specialist or primary care provider for the management of childhood asthma.

### Limitations

A limitation of this analysis is that the survey was conducted online and for English-speaking participants. Therefore, families who do not have internet access or are non-English–speaking are not represented. Future studies are needed for these populations. Survey response options for providers who gave AAPs were mutually exclusive; there may have been parents who received an AAP both from their PCP and specialist. If parents received an AAP from both sources, this may have influenced their self-efficacy and feeling of helpfulness. Further, participants were asked about their perceptions of the AAP’s helpfulness rather than the number of actual missed school days and workdays; measures of helpfulness were not validated, and asthma control and AAP use were not measured with validated instruments. However, parents’ perceptions of not missing work and school may still offer a relevant measure of a sense of disruption due to asthma. Future studies could consider examining whether parents with higher parental self-efficacy and increased confidence in their child’s asthma care are associated with a decrease in measured office visits and sick days off school. Direct, quantified measurement of asthma control and AAP by specified users could also be evaluated.

### Conclusions

Overall, this study supports the use of AAPs as a valuable tool associated with numerous practical daily benefits that improve the management of pediatric asthma. Further, these benefits were consistently reported across parents who received AAPs from different provider types and who had varied socioeconomic backgrounds. These findings reinforce the recommendations of the Centers for Disease Control and Prevention and the American Academy of Pediatrics to provide AAPs to children. AAPs should be offered as standard asthma care for children by all providers. Technology advancements can be used to further improve asthma control for children, such as online or app-based AAPs. The development and promotion of easily sharable AAPs with school nurses, coaches, and caregivers through laptops and cell phones would help eliminate barriers for families and children with asthma from fully participating in and enjoying school, family, and childhood activities.

## Abbreviations

AAP: Asthma Action Plan
PCP: primary care provider
